# Bridging Basic Science with Cardiac Surgery: The Bristol Heart Institute Experience

**DOI:** 10.3389/fsurg.2015.00031

**Published:** 2015-07-22

**Authors:** Costanza Emanueli, Gianni D. Angelini

**Affiliations:** ^1^Bristol Heart Institute, University of Bristol, Bristol, UK

**Keywords:** cardiac surgery, vascular surgery, basic science, translational research, regenerative medicine, biomarkers

## A Basic Scientist Perspective

I am honored to serve as an associate editor for Frontiers in Surgery, Heart Surgery Subsection. My current basic and translational research is in the field of non-coding RNAs, angiogenesis therapy, and innovative cellular products for cardiovascular therapeutics^1^. The main translational focus is on patients with ischemic disease undergoing cardiac and vascular surgery. My team applies basic science approaches and works on clinical samples and databases with the aim of advancing the mechanistic understanding of diseases that lead to the need for surgery, to identify potential markers for patient risk stratification, and to offer surgical patients novel regenerative medicine approaches that synergize with standard care. I have worked in the cardiovascular field for more than 20 years, the last 10 years as a next door neighbor to an excellent academic cardiac surgery unit (led by my co-author)^2^. Notwithstanding and possibly surprisingly enough, it is only in the last few years that I started to understand what cardiac surgeons really do in their clinical practice and especially what kind of research is of relevance to them. For many basic scientists, the translational goals are a bit naïve and dreamy (“mending a broken heart”) and centered on our own research, developed largely independently from the potential final users (surgeons and other frontline clinicians), who in our imagination, should be able to deliver a clinical trial to test our proposed therapeutic remedy based on work in cells and small animal models. Cardiac surgery is ignored by most of us, who rarely get in touch with surgeons, especially the rare kind that want to collaborate and offer something more than spare clinical samples in return for authorship. However, once a good rapport is established, the possibilities for rewarding collaboration are high and I consider my last years of research among the most exciting, productive and fun I ever had. The topics for collaboration are large and below we will discuss some of them. For the readers of this journal, I think it might be interesting to understand my perspective as a basic scientist (other basic scientists can of course disagree) on some issues that might compromise many of these successful interactions. Prejudices, stereotypes, and ignorance from both ends are not helpful, but unfortunately common. One of the most frequent is that *cardiovascular surgeons are just plumbers and they should not do research*. This is something that I have heard more than once, including from the surgeons themselves. I think this is simply wrong and based either on ignorance or self-deprecation leading to justification for an “easier” professional life. Cardiac surgeons are medically qualified and usually some of the most ambitious clinicians; it is unlikely that working as a surgeon will reduce their intellectual capacities. However, it is true that the surgeons’ clinical duties are mentally and physically demanding and emergencies are often managed at antisocial hours. Consequently, when possible the surgeons need to switch off (this also applies to basic scientists) for their own sanity and for the safety of their next patient. This does not mean that they (if they are good academics and collaborators) will not switch on again in time to complete some agreed tasks. Moreover, in the academic environment, we should insist that clinicians (including the junior ones) are given more opportunities for research-protected time and provided with support first and foremost from a clinical trial unit. Clinicians, on the other hand, not infrequently consider their basic science counterparts *as belonging to a “B class” in the Faculty of Medicine*: this is not helped by a distinction in remuneration (“clinical and non-clinical salary scales”) paid to equivalent academic figures. However, in the changing world, sacrifices comparable to their clinical colleagues are required from high profile, basic scientists, including being more and more often “on call” for deadlines on any sort, in the first instance grant applications, which must be met and successful to keep the research and labs running. The basic scientists’ managerial, ethical, and legal responsibilities have also dramatically increased. To succeed in this increasingly competitive high-pressure environment, we have to develop the classical “surgeon thick skin.” I have to come clean and say that in Bristol, I have never felt “discriminated against” by my surgeon collaborators for being a basic scientist. Rather, I feel they have an admiration for my different kind of work (as I have for theirs) and welcome my approach to improve their clinical studies with a healthy mutual respect essential in building fruitful collaborations. This reciprocal respect does also apply to the other basic scientists and this culture was proven when my surgeon co-author teamed up with Professor Andrew Newby (his British Heart Foundation basic scientist Chair counterpart) to establish the Bristol Heart Institute^3^ in 1995. I share the vision of this research model, which is grounded on the dialog between scientists and clinicians with different research backgrounds, and often involves leaving our comfort zone (research niche) to explore new avenues. This is the only way to identify the “real” medical needs of the moment and design research that has the potential to lead to clinical applications. I truly believe that only openness, altruism, and collaboration with like-minded individuals with different backgrounds will enable us to advance the basic science knowledge required to impact on clinical practice in cardiovascular surgery.

## And Now, Let Us Talk Science!

### Zooming in the human diseased heart

Cardiac and vascular surgery offers the opportunity to perform fundamental research using a range of samples taken from patients. Cardiologists can give you some blood and urine (and rarely myocardial biopsies), but leftover tissue from surgery can provide precious human material. This offers the opportunity to develop fundamental research in patients. Functional studies on cells prepared from human samples might reduce the use of animal models and concentrate them mainly to proof-of-principle for efficacy, preclinical safety, and feasibility studies. We basic scientists are aware of the limitations of animal models that we use in our “research practice.” It is not only a common knowledge that sophisticated genetic mouse models favor publications in the very top scientific journals but also true that they do not mimic human pathology well enough. Moreover, the cost and ethical issues linked to animal research are growing exponentially. It may be argued that preclinical work on large animals would be a better investment, but this often requires clinical knowledge, particularly when conducting interventions modeling the clinical scenario. Examples of cardiac surgery-relevant animal models are the pediatric ones created by our colleague Professor Caputo (another associate editor of the *Frontiers in Heart Surgery* journal), who has succeeded in mimicking replacement of the pulmonary artery and valve in young piglets to test the growth capacity of new tissue engineered conduits (see http://www.bristol.ac.uk/cardiovascular/people/massimo-caputo/index.html: video 2 JT award and https://www.dropbox.com/s/8l1rc8z2vr1s7h2/JT%20award%20video%202.m4v).

## Omics and System Biology for Defining Therapeutic Targets and Novel Biomarkers

Cardiac surgery offers the opportunity to analyze in parallel changes induced by pathologies of the myocardium (or other organs or tissues, such as heart valves and thoracic vessels) and biological fluids. Using the new *omics* possibilities and good bioinformatics and systems biology, the opportunity exists not only to identify new clinically relevant therapeutic targets for validation in basic science investigations using cells and research animals, but also to define circulating/urinary biomarkers that provide information about expressional changes in the heart and link them to patients’ clinical profiles. In cardiac surgery, the most needed biomarkers are those enabling prediction of post-surgical acute complications, like acute kidney injury (1), and long-term outcomes, such as aneurysm evolution, in many bicuspid aortic valve surgical patients (2). This knowledge would facilitate monitoring and treatment and also allow for better-designed randomized clinical trials (RCTs) using the new biomarkers as surrogate end-points.

## Something for the More Optimistic: Gene Therapy, Stem Cells, and Tissue Engineering

It is the dream of all basic and clinical scientists to find new solutions to offer better therapies for patients. The surgeons know that they can further strive to improve their surgical techniques, but some of their interventions will remain palliative or not completely resolutive. Pediatric cardiac surgeons, in particular, suffer the frustration of having to re-operate on their patients again and again as they grow to adulthood whereas the conduits and materials used for the surgical correction do not. The currently available drugs cannot “*regenerate, repair, replace*” death or missing parts of the heart, valves, and blood vessels. Regenerative medicine was thought to be the answer to fill this gap. For cardiovascular scientists, this started with angiogenesis gene therapy and went on with stem cell transplantation with the hope to growth new blood vessels and even myocardial muscle (3, 4). After the initial enthusiasm, gene therapy has been in crisis for a while because it could not easily deliver the clinical success we all hoped for (5, 6). We believe that the efforts of basic scientists to improve viral and non-viral vectors and the newly identified non-coding RNA targets (especially microRNAs) will “regenerate” interest and boost more preclinical and early clinical studies in the area of therapeutic angiogenesis, cardiovascular protection and regeneration, aneurysms, and vascular graft failure.

Stem cells from different sources have been discovered and tested in animal models. In the clinic, mostly bone marrow-derived cells have been trialed, with mixed success (3, 4). Stem cells and cardiac surgery have not mixed much together so far, even if small RCTs have been done or are ongoing, including at our institution (7–11). We have always been fascinated by the potential of stem cells for cardiovascular therapies and we have contributed significantly to this field (12–15). At the same time, we remain a little skeptical that the approaches used so far in cardiovascular clinical trials, largely based on transplantation of different types of stem cells administered in a liquid solution and “left to their destiny” in an hostile environment, will produce any sustained therapeutic effect. In fact, most of the adult cell populations considered for clinical use have shown a poor engraftment capacity and elicit mainly paracrine actions before dying or being washed out. While we believe that basic stem cell research including that on cardiac surgery-derived samples should continue and we are pursuing it extensively, a different focus of stem cell research is needed to fulfill cardiovascular regeneration promises. We believe that the new frontier is to use such stem cells together with clinical-grade matrices to build tissue structures. This is not only relevant in congenital heart surgery in infants and children but also for valve replacement in adult patients. Finally and more futuristically, tissue engineering should deliver off the shelf myocardial structure to be used to replace large scars and congenitally lacking myocardium in surgical patients. This can be corroborated by the recent advances in protocols for *in vitro* and even *in vivo* direct reprograming of fibroblasts to myocytes (16, 17).

## Basic Science “Service” to Cardiac Surgery

Once there is a good team work ethos, basic scientists can also help modernize and energize the more classical surgical studies by providing expertise to apply new technologies and refining the lab analyses to investigate “surgical questions.”

In conclusion, we have focused on our direct experience of harmonious collaborations between basic scientists and a cardiac surgery unit. This reflects our vision of a multidisciplinary team capable of bridging novel fundamental science with clinical practice to synergize the research potential at both ends. This is the ethos and culture we wish to pass to the young generation of basic and clinical trainees in Bristol and elsewhere. The clinicians can identify the medical needs and priorities, the basic scientists provide the understanding of the mechanisms of action and “new ideas” and together we can propose and test new solutions for the benefit of our patients.

## Conflict of Interest Statement

The authors declare that the research was conducted in the absence of any commercial or financial relationships that could be construed as a potential conflict of interest.

## Footnotes

^1^http://www.bristol.ac.uk/cardiovascular/people/costanza-emanueli/index.html

^2^http://www.bristol.ac.uk/cardiovascular/people/gianni-d-angelini/index.html

^3^http://www.ukcrcexpmed.org.uk/BristolCVBRU/Pages/FacilityWelcome.aspx
